# Genetics of the congenital absence of the vas deferens

**DOI:** 10.1007/s00439-020-02122-w

**Published:** 2020-02-05

**Authors:** Eric Bieth, Safouane M. Hamdi, Roger Mieusset

**Affiliations:** 1 Service de Génétique Médicale, Hôpital Purpan, CHU, 31059 Toulouse, France; 2 Service de Biochimie, Institut Fédératif de Biologie, CHU, 31059 Toulouse, France; 3 EA3694 (Groupe de Recherche en Fertilité Humaine), Université Toulouse III, 31059 Toulouse, France; 4 Département d’Andrologie (Groupe Activité Médecine de la Reproduction), CHU, 31059 Toulouse, France

## Abstract

Congenital absence of the vas deferens (CAVD) may have various clinical presentations depending on whether it is bilateral (CBAVD) or unilateral (CUAVD), complete or partial, and associated or not with other abnormalities of the male urogenital tract. CBAVD is usually discovered in adult men either during the systematic assessment of cystic fibrosis or other *CFTR*-related conditions, or during the exploration of isolated infertility with obstructive azoospermia. The prevalence of CAVDs in men is reported to be approximately 0.1%. However, this figure is probably underestimated, because unilateral forms of CAVD in asymptomatic fertile men are not usually diagnosed. The diagnosis of CAVDs is based on clinical, ultrasound, and sperm examinations. The majority of subjects with CAVD carry at least one cystic fibrosis-causing mutation that warrants *CFTR* testing and in case of a positive result, genetic counseling prior to conception. Approximately 2% of the cases of CAVD are hemizygous for a loss-of-function mutation in the *ADGRG2* gene that may cause a familial form of X-linked infertility. However, despite this recent finding, 10–20% of CBAVDs and 60–70% of CUAVDs remain without a genetic diagnosis. An important proportion of these unexplained CAVDs coexist with a solitary kidney suggesting an early organogenesis disorder (Wolffian duct), unlike CAVDs related to *CFTR* or *ADGRG2* mutations, which might be the result of progressive degeneration that begins later in fetal life and probably continues after birth. How the dysfunction of *CFTR*, *ADGRG2,* or other genes such as *SLC29A3* leads to this involution is the subject of various pathophysiological hypotheses that are discussed in this review.

## Background

### Anatomy

**Fig. 1:**
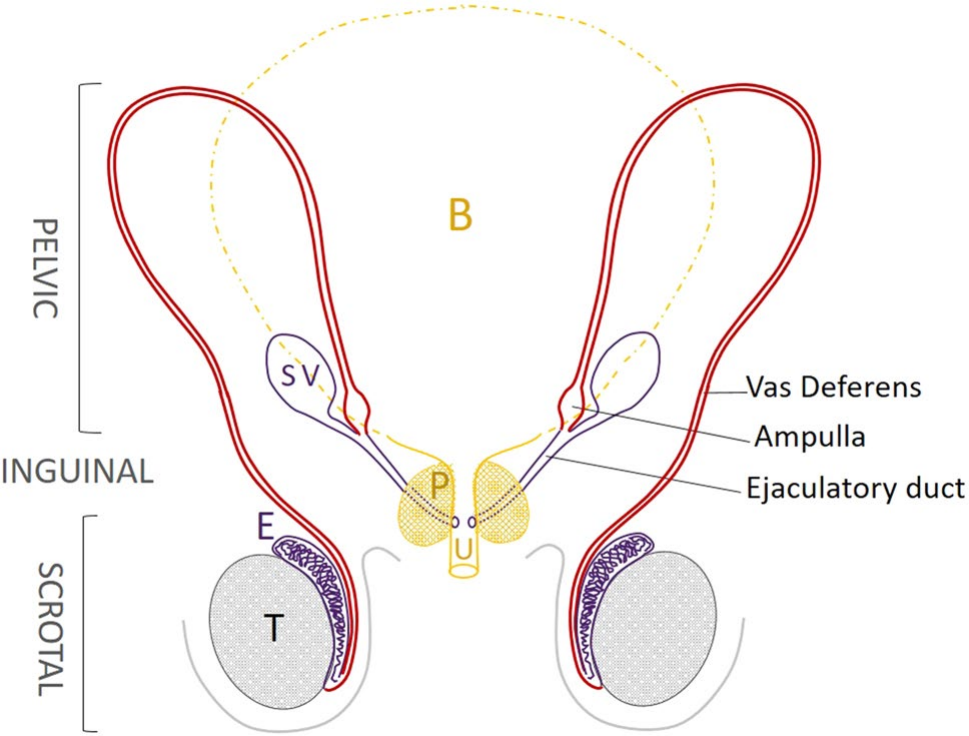
Anatomy of the adult vas deferens. The proximal part of the vas deferens (VD, in red) that follows the epididymis (E) is located in the scrotum, easily accessible for palpation. The VD then travels through the inguinal canal and then into the pelvis in a retroperitoneal position. The dilated terminal part of the VD is called the ampulla of the vas deferens. The ejaculatory duct follows the ampulla of the vas deferens with the end of the seminal vesicle and opens in the posterior surface of the prostatic urethra (U). *B* bladder, *P* prostate, *SV* seminal vesicle

The vas deferens (VD) is the tubular structure that connects the epididymis (E) to the ejaculatory duct (Fig. 1). The VD follows the epididymal duct and ends in dilation, the ampulla of the vas deferens. The organ epididymis includes three successive regions, the caput, corpus, and cauda. The head is mainly filled by the efferent ductules; the body and the tail by a single epididymal duct. The VD is part of a pair symmetrical set, the seminal duct which includes the epididymal duct, the VD, and the ejaculatory duct with which a gland, the seminal vesicle, is directly anastomosed. This set is derived from the mesonephric ducts also called Wolffian ducts.

### Embryology

**Fig. 2:**
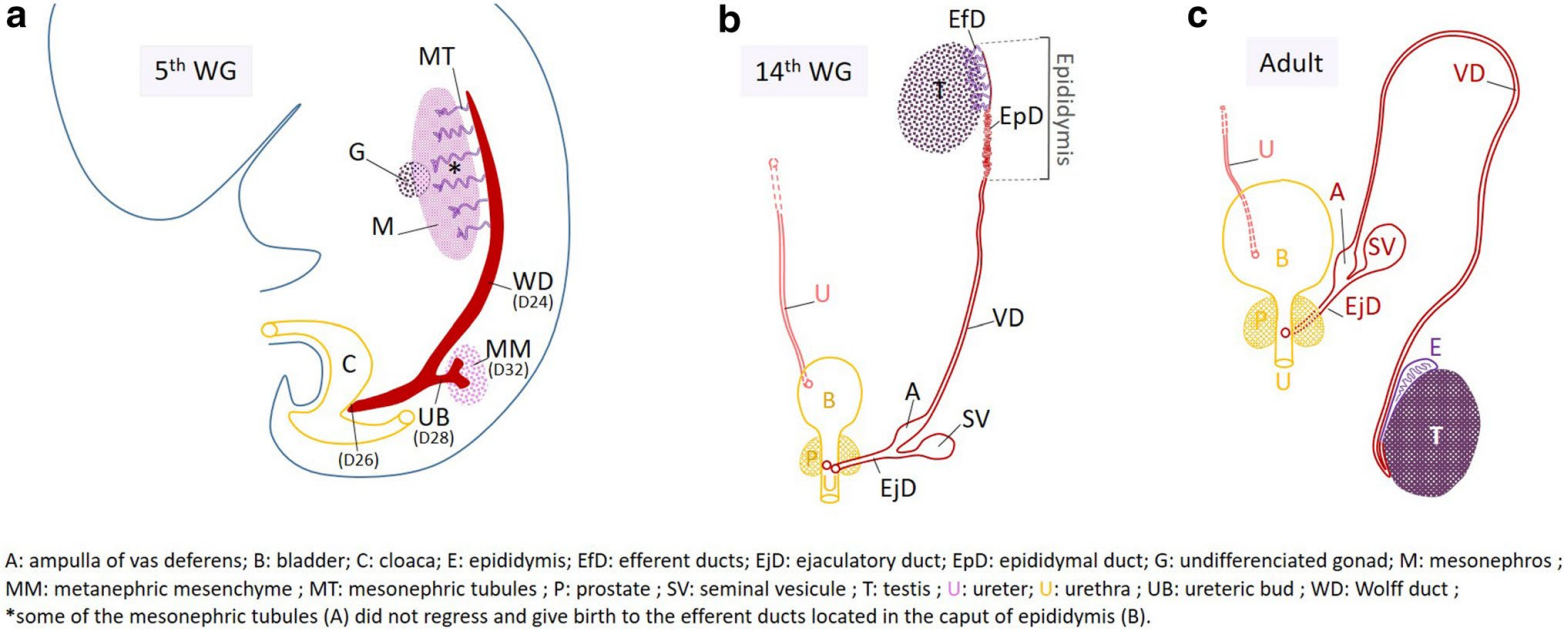
Brief history of the development of the Wolffian duct in males. **a** Apparition and elongation of the WD (cord of cells indicated in red) during the 5th week of gestation (WG). **b** WD derivatives (indicated in red) at 14 WG. The efferent ductules (EfD, in purple), derived from the mesonephric tubules, occupy the head of the epididymis, the body and tail of which contain the epididymal duct (EpD) which is followed by the vas deferens (VD). The ascent of the mature kidney leads outside the image which only shows the right ureter (U). **c** Testis and genital male tract in adulthood

Most components of the genitourinary system are derived from the intermediate mesoderm (Fig. 2a) that gives rise to a pair of a solid cord of cells called nephrogenic cords in the 3rd week of gestation (WG3). The latter induce the sequential rostrocaudal formation of pronephros, mesonephros, and metanephros. Mesonephros and Wolffian ducts (WD) differentiate during WG4. More precisely, the nephrogenic cords elongate by cell proliferation from its caudal end towards the ventrolateral surface of the cloaca with which they merge at day 26 (D26) at the posterior surface of the urogenital sinus. After fusion, the cords hollow out from a lumen and become the WD (Fig. 2a). At the end of GW4, the epithelial cells of each WD produce an outgrowth in their caudal part known as the ureteric bud (UB). During WG5, the UB enters the adjacent intermediate mesoderm, called metanephric mesenchyme (MM), in response to inductive signals from the MM (Jain and Chen 2018). In the mesonephros, mesonephric tubules develop and their distal end opens into WDs that lead the urine secreted by the mesonephros from WG6 to 10 into the future bladder. The mesonephros is the functional kidney as of WG 6–10. The ensuing gonadal differentiation into testes (WG7) and the secretion of androgens by the Leydig cells enable the conservation and differentiation of WDs. During WG4 and WG5, the inclusion of WDs in the posterior wall of the future bladder occurs by a process of exstrophy (Fig. 2a). The result is an opening of the ureteric buds in this posterior surface which is almost that of their initial implantation site, while the WD orifices descend to the future prostatic urethra (Jin et al. 2016). The cephalic region of the WD gives rise to the epididymal duct (Domeniconi et al. 2016) that occupy the body and tail of the epididymis, and to the median region to the VD (Fig. 2b). The caput of epididymis shelters the efferent ductules which derive from the mesonephric tubules; they ensure the connection of each seminal duct with the testis. In WG 10–12, a new bud appears in the caudal portion of the WD which gives rise to the seminal vesicle and establishes the ampulla of the vas deferens upstream and the ejaculatory duct downstream (Brewster 1985). During this period, the prostate develops from lateral buds of the future prostatic urethra. Development of the VD results in a long tube following the end of the epididymis and that extends to the ampulla. Finally, the WD produces the following derivatives (indicated in red in Fig. 2b, c): the epididymal duct, the VD with the ampulla, the ejaculatory duct, and the seminal vesicle. The prenatal migration brings the testis into the scrotum, pulling along the epididymis and the proximal part of the vas deferens which is accessible by palpation during adulthood (Fig. 2c).

### Physiology

**Fig. 3:**
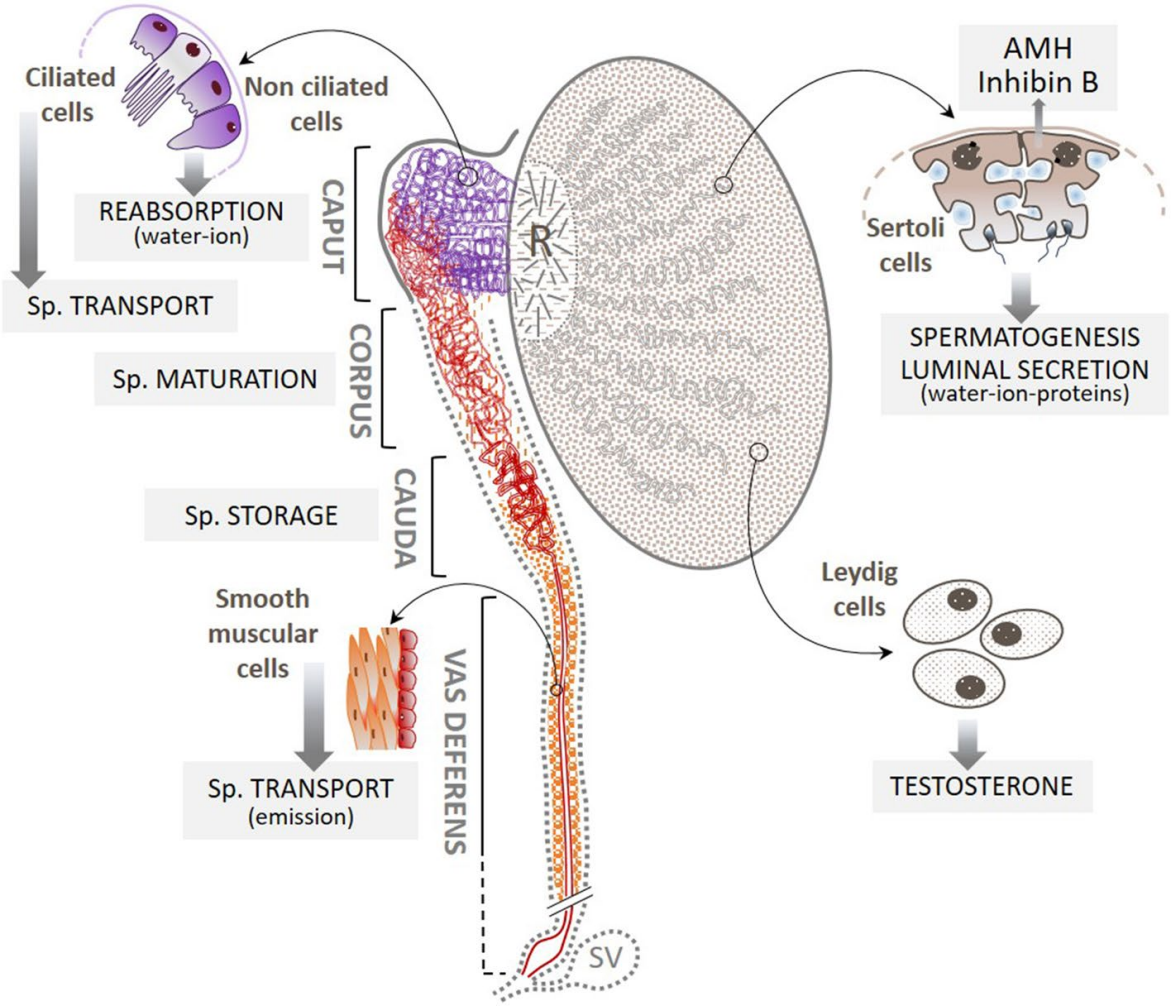
Main functions of the efferent ductules, the epididymal duct, and the vas deferens. The Sertoli cells secrete a fluid in the lumen of seminal tubules which converge towards the rete testis (R); about 90% of it is reabsorbed by the non-ciliated epithelial cells of the efferent ductules (in purple), leading to a concentration of spermatozoa. These are then transported into the epididymal duct where some of the epithelial cells contribute to the creation of a new intraluminal fluid that bathes the spermatozoa (Sp.). These cells are also at the origin of the sperm maturation process. Sperm are continuously produced in the epithelium of the seminal tubules and are stored in the tail of the epididymal duct. Part of this stock is mobilized during ejaculation (emission phase) and propelled into the vas deferens, where the layers of smooth muscle cells enable these spermatozoa to be rapidly transported into the posterior urethra via the ejaculatory duct. Gray dotted highlighting indicates structures that, in addition to the vas deferens, may be absent in CAVD. *SV* seminal vesicle

In the testis, spermatozoa are transported by the intraluminal fluid of the seminiferous tubules secreted by the Sertoli cells, into the rete testis with which the efferent ducts (EfDs) are connected (Fig. 3). The EfDs reabsorb 90% of this intraluminal fluid. This process performed by the non-ciliated cells of EfD epithelium results in a concentration of spermatozoa that are then exposed to a new fluid secreted by the epithelial cells of the epididymal duct. The latter ensures the transport of spermatozoa by intraluminal hydrostatic pressure, their maturation (acquisition of fertilizing capacities and progressive mobility properties), immunoprotection, storage (Sullivan and Mieusset 2016), and, finally, expulsion into the VD by contraction of the myoid cell layers in the cauda of the epididymis. The VD transports spermatozoa and, thank to the secretory characteristics of its main epithelial cells, it ensures the survival and mobility of spermatozoa.

### Definition of the CAVD (different forms), history, and clinical diagnosis

**Fig. 4:**
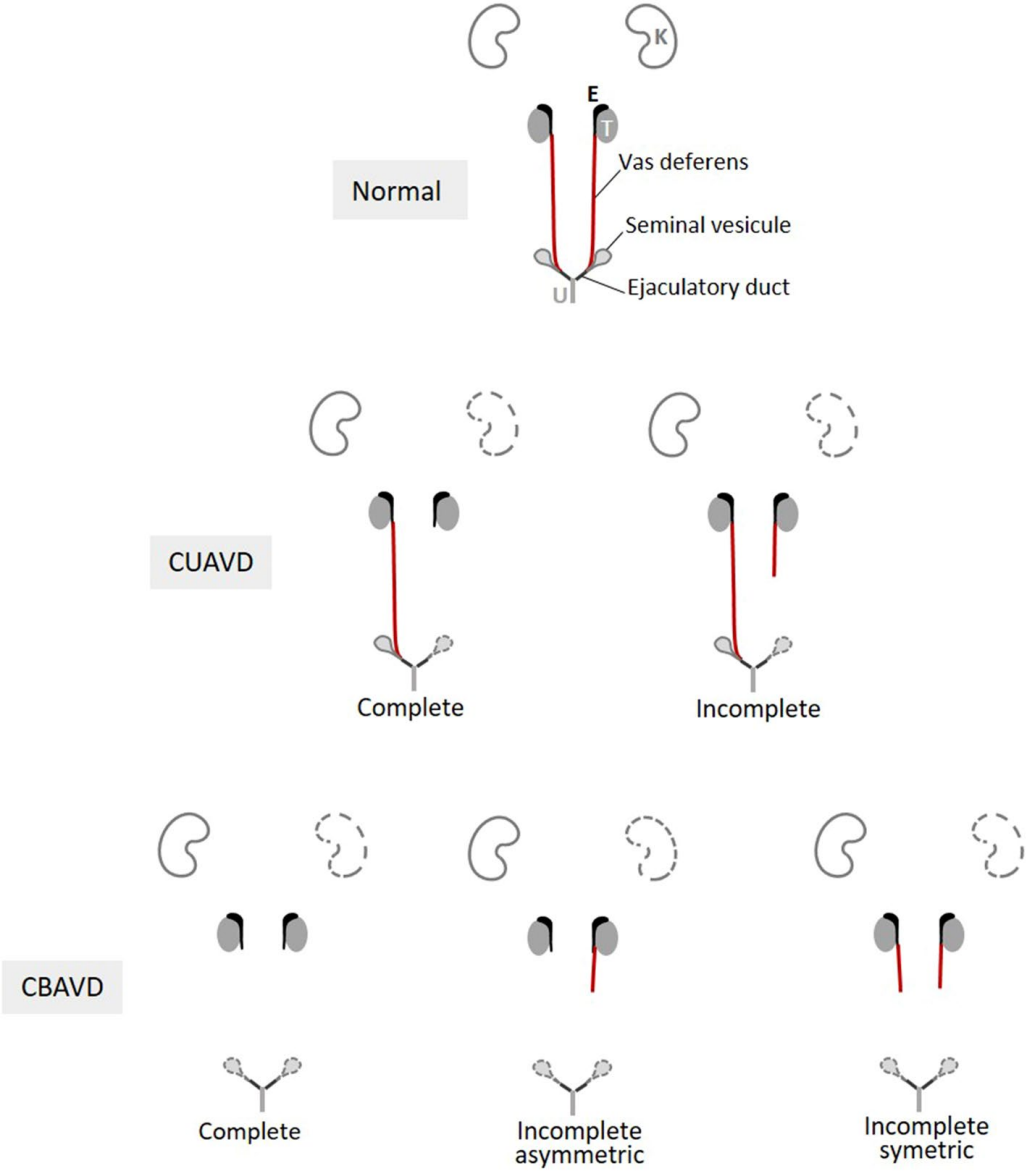
Presentations of the morphological anomalies of CBAVD and CUAVD. Three presentations of CBAVD and two presentations of CUAVD are morphologically identifiable based on the absences reported by imaging of the vas deferens alone (indicated in red). Possible absences of a kidney and/or one or both seminal vesicles are represented by the organs with dotted lines. Other potentially associated anomalies such as absences from the body and/or tail of the epididymis, the ampulla of the vas deferens and the ejaculatory ducts are not taken into account. *E* epididymis, *K* kidney, *T* testis, *U* urethra

The absence of vas deferens was first noted on a corpse in 1755 by Hunter, a renowned Scottish surgeon and anatomist (Hunter 1786). However, it was not until the mid-twentieth century that urologists began to consider CAVD as a clinical entity responsible for male infertility (Nelson 1950). The numerous clinical observations that followed now make it possible to distinguish five anatomical presentations of CAVD (Fig. 4): three for bilateral absences (CBAVD) and two for unilateral absences (CUAVD). This diversity of phenotypes makes it difficult to elucidate the etiopathogenic mechanisms, especially since morphological anomalies that affect two other organs, the seminal vesicle (SV) and the kidney may or may not be associated with CAVDs. SV size abnormalities (hypotrophy, atrophy, dilation) or the absence of SVs have been reported with variable frequencies, probably due to differences in the detection methods used (Goldstein and Schlossberg 1988; Casals et al. 2000; Schlegel et al. 1996). Overall, bilateral SV anomalies appear to be twice as frequent in CBAVD (50% vs 25%), while unilateral SV anomalies are mainly reported in CUAVD (80%) where they are generally ipsilateral (Schlegel et al. 1996). However, these data on CUAVD mainly concern men with azoospermia. In contrast, more studies have been conducted on the association of CAVDs with renal abnormalities. As early as 1870, a Swiss surgeon, Reverdin, described unilateral renal absence (URA) in a man with CUAVD and an ipsilateral absence of SV (Reverdin 1870). Since then, numerous studies (Schlegel et al. 1996; Casals et al. 2000; Weiske et al. 2000; Patat et al. 2016; Mieusset et al. 2017, 2020) have confirmed that URA is found with CAVDs at a high rate of 5–40%, while the prevalence of URA at birth is nearly 1,000 times lower (4/10,000) (Laurichesse Delmas et al. 2017). It is important to note that URA is found two-to-three times more frequently in patients with CUAVD (20–40% of the cases, with URA being predominantly ipsilateral) than in those with CBAVD (5–10% of cases).

### Prevalence and diagnosis of CAVDs

The prevalence of CAVD (all forms combined) in men is estimated to be approximately 0.1%, but this figure might be underestimated, because unilateral forms of CAVDs in asymptomatic fertile men are usually only diagnosed during vasectomy (Miller et al. 2016). CAVD is found in 1–2% of infertile men (Jequier et al. 1985; Weiske et al. 2000). There are three major circumstances in which CAVD is diagnosed; either in men with cystic fibrosis (CF, MIM#219700) or other genetically related conditions called CFTR-RD (for CFTR-related disorders) for which they have symptoms; or in apparently healthy men who are referred for infertility due to azoospermia; or in asymptomatic fertile men with CAVD discovered by chance (e.g., during vasectomy). CBAVD is present in almost all boys with CF (Kaplan et al. 1968), a severe autosomal recessive disorder typically manifested by exocrine pancreatic insufficiency, progressive lung disease with a poor prognosis, and abnormally high levels of chloride in sweat (Farrell et al. 2017). Cystic fibrosis is usually diagnosed at birth (screening) or in the early years, but in exceptional cases, it is the exploration of infertility that leads to the diagnosis. In this review, we will distinguish two clinical forms of CBAVD: CBAVD associated with symptoms belonging to the cystic fibrosis clinical spectrum (CF-CBAVD) and isolated CBAVD (iCBAVD). In all cases, the diagnosis of CAVD is clinical. For a long time, it was based solely on palpation of the VD, that is to say, on their intrascrotal portion: the diagnosis was positive if this portion was absent, and negative if it was present. At present, in addition to palpation, the diagnosis is based on ultrasound imaging (scrotal and transrectal), which is the gold standard: ultrasound objectivizes the presence (partial or complete) or absence of VDs and specifies the status of the ampulla of the vas deferens and the SVs (Schlegel et al. 1996; Daudin et al. 2000). In case of CBAVD associated with bilateral absence of SV, the biological warning sign is a non-pathognomonic triad: hypospermia (semen volume < 1.5 ml), acid pH (< 7.0) and levels of seminal plasma biochemical markers lower than the lower references values (fructose < 13 μmol/ejaculate; GPC < 2 µmol/ejaculate). This seminal picture is not as straightforward if one or both SVs are present. In all cases, azoospermia is present. In case of CUAVD, the diagnosis is very often made when azoospermia is present (by acquired obstruction of the only permeable seminal duct), but it should be emphasized that in infertile couples, men with CUAVD are mainly non-azoospermic (Mieusset et al. 2020). Indeed, in the absence of contralateral obstruction in case of CUAVD, the semen is more or less rich in spermatozoa.

## Genetics of CAVD

### Methodological approaches

The genetics of CAVDs has been historically linked to CF since 1968 when Kaplan et al. showed that almost all men with CF have obstructive azoospermia (OA) due to CBAVD (Kaplan et al. 1968). The concept that isolated forms of CBAVD and cystic fibrosis are linked to the same gene was confirmed shortly after the *CFTR* gene was identified in 1989 (Dumur et al. 1990; Anguiano et al. 1992). However, it was soon noted that 20–40% of the CBAVDs cases were not linked to *CFTR* mutations, suggesting the possibility of genetic heterogeneity (Culard et al. 1994; Chillón et al. 1995). Nonetheless, for 25 years, the genetics of CAVD remained restricted to *CFTR*. The fact is that the study of the genetic determinism of human infertility has long been restricted by specific methodological constraints, with the traditional family-based approaches by linkage analysis often being inapplicable, particularly in the context of male infertility due to psychosocial and cultural barriers. Therefore, until recently, the genetic markers commonly used in clinical practice to explore a non-obstructive azoospermia (NOA) were limited to chromosomal abnormalities such as Klinefelter’s syndrome and Y chromosome microdeletions (Krausz 2011). However, over the last decade, the advent of next-generation sequencing (NGS) has enabled the development of powerful approaches based on whole-exome sequencing (WES) or whole-genome sequencing (WGS) and whole transcriptome analysis. Approximately 20 genes involved in monogenic forms of NOA have recently been identified (reviewed by Ghieh et al. 2019). For almost all of these genes, causal mutations, all recessive, have been identified in consanguineous families (Yang et al. 2018). On the other hand, to our knowledge, exceptional family cases of OA have never been observed in a context of consanguinity, a limitation that partly explains why, despite the facilities of the new genomic approaches, the number of new genes identified in CAVD has been far lower. Nevertheless, in recent years, two major complementary approaches have contributed to the identification of candidate genes; those based on the study of individual genomes and those based on seminal duct cell transcriptome analysis, mainly of the epididymis. The first led to the establishment of relevant correlations between iCBAVD and point mutations of the *ADGRG2* gene identified by WES analysis (Patat et al. 2016; Khan et al. 2018), as well as copy-number variations of the *PANK2* and *SLC9A3* genes identified by array-based comparative genomic hybridization (array-CGH) analysis (Lee et al. 2009). Transcriptomic approaches using the cDNA microarray or RNA sequencing have succeeded in targeting many functional candidate genes, particularly genes whose expression is restricted to cells of the seminal duct or whose expression profile is specific to certain parts of the seminal duct (Browne et al. 2016). Several of these candidate genes such as *ADGRG2* and *SLC9A3* have been validated in knockout mice and their physiology explored in this animal model (Davies et al. 2004; Wang et al. 2017). Recently, an integrative multi-omic approach that combines WGS, whole DNA methylome analysis, and RNA sequencing has led to the identification of two new candidate genes, *SCNN1B* and *CA12,* in an individual with iCBAVD (Shen et al. 2019). However, despite these advances, there is still no genetic diagnosis for at least a quarter of the CAVDs, most of which are CUAVDs. Until now, the hypothesis that these unexplained forms of CAVD are not the result of simple genetic variations has been little explored. It is foreseeable that in the future, the new methods for studying the epigenome and the power of bioinformatic tools will make it possible to specify the role of epigenomic regulation in the occurrence of these isolated CAVDs. However, this approach will be all the more successful if it is applied to cohorts of adequate size composed of perfectly phenotyped CAVD patients of a homogeneous ethnic origin.

### Genes implicated in CAVD

While the genetic determinism of NOAs is characterized by significant genetic heterogeneity with more than 30 genes identified (SPGF [MIM#258150]), that of OAs is limited to very few genes (Ghieh et al. 2019). Therefore, it is established that approximately three-quarters of the Caucasian cases of CAVD are linked to anomalies in two genes: *CFTR* for the majority of cases and *ADGRG2* for a minority (Patat et al. 2016). Other genes such as *SLC9A3* might be involved in some iCBAVDs but also epigenetic or environmental factors with very different physiopathological roles.

*CFTR* (MIM#602421) was identified by positional cloning in 1989 by Riordan et al. (1989) ending several years of competitive research to discover the sole gene responsible for CF. Approximately half of the CF patients of northern European descent are homozygous for a deletion of three base pairs (NM_000493.3:c.1521_1523del), resulting in the loss of phenylalanine 508 (NP_000483.3:p.Phe508del, legacy name: F508del). On average, 1 in 40 individuals in the Caucasian population is heterozygous for p.Phe508del mutation, making it one of the most frequent human pathogenic mutations (Kerem et al. 1989). *CFTR,* which covers 250 kb on the long arm of chromosome 7 in 7q31.2, contains 27 coding exons and produces several transcripts, only one of which, a 6.1-kb mRNA, codes for a functional protein of 1,480 amino acids called CF transmembrane conductance regulator (CFTR). CFTR is a glycosylated transmembrane protein expressed at the apical membrane of many epithelial cells where it functions mainly as a cAMP-regulated chloride channel. Many studies have shown that CFTR is involved in the regulation of several ions transporters including sodium channel (ENacs), chloride/bicarbonate exchangers, protons exchangers (Na^+^/H^+^), and water channels (aquaporins). Therefore, CFTR-dependent physiological processes play a crucial role in maintaining the homeostasis of ions, pH, and water in secretory epithelial fluids (Choi et al. 2001). In 3 decades, more than 2000 mutations have been reported in *CFTR* (https://www.genet.sickkids.on.ca/), but less than a quarter are classified as pathogens (https://www.cftr2.org/) based on correlations with CF or other conditions that often have a less severe prognosis, limited to a single organ such as the bronchi (disseminated bronchiectasis, MIM#211400), pancreas (chronic pancreatitis MIM#167800) or the vas deferens (congenital bilateral absence of vas deferens, MIM#277180). These conditions that do not meet all the criteria for cystic fibrosis but are related to CFTR dysfunction have been grouped under the generic term CFTR-RD (Bombieri et al. 2011). All regions of *CFTR* can be affected by disease-causing mutations including promoter regions and deep intronic regions (Feng et al. 2019; Bergougnoux et al. 2019). Depending on their effects on the biogenesis and functions of CFTR, pathogenic alleles are classified into two main categories: CF-causing variants (also called "severe") which, in the homozygous state, are always associated with CF and non-CF-causing variants which have never been observed in CF patients and which are, therefore, mistakenly called "mild" alleles. A minority of CF-causing alleles have been observed in variable clinical forms of more or less severe cystic fibrosis in which pancreatic function is often preserved. The pathogenicity of these alleles called VCC (for variants of varying clinical consequence) might depend on rarely known genetic factors such as *cis* association with complex alleles or unknown non-genetic factors. Unlike CF-causing variants, non-CF-causing variants cause an incomplete CFTR dysfunction. Depending on the organ, if the residual activity of CFTR is too low to maintain homeostasis, a CFTR-RD may appear. Therefore, subjects with CFTR-RD generally carry a non-CF-causing variant most often combined in *trans* with a CF-causing variant or, more rarely, with another non-CF-causing variant. These alleles are sometimes referred to as CFTR-RD-causing alleles.

*ADGRG2* (MIM#300372) located in Xp22.13 is composed of 29 exons that produce approximately ten transcripts, the longest of which has an open-reading frame of 3.1 kb (covers exons 3–29) that encode for the adhesion G protein-coupled receptor G2 (ADGRG2). Its cDNA was initially cloned in 1997 by Osterhoff et al. (1997) after differential screening of a library of human epididymal cDNA cells in which this clone, named HE6 (for human epididymis-specific protein 6) was vastly represented. With a deduced 1017 amino acids’ sequence and its seven highly preserved transmembrane domains, the HE6 protein belongs to the G *protein-coupled receptor* (GPCR) superfamily in which it was originally referred to as GPR64. The structure and autocatalytic property of the extracellular part of HE6/GPCR64 led to its final classification in the G sub-family of the adhesion GPCR (aGPCR) (Hamann et al. 2015). ADGRG2 is a highly glycosylated protein almost exclusively and highly expressed in the proximal part of the male seminal ducts (https://proteinatlas.org), precisely in the epithelium of the efferent ductules and the initial part of the epididymal duct. ADGRG2 immunolabeling is particularly strong in the stereocilia of the main epididymal cells and in the microvilli of the non-ciliated cells of the efferent ductules where 90% of the fluid secreted by the testis is reabsorbed (Kirchhoff et al. 2008; Patat et al. 2016). The involvement of ADGRG2 in this process was initially suggested by HE6/GPR64 knockout (targeted disruption) in mice, which, in hemizygous males, results in an accumulation of fluid in the testis and sperm stasis in the efferent ductules leading to an obstructive infertility phenotype (Davies et al. 2004). ADGRG2 is an orphan receptor with unknown natural ligands and partially elucidated signaling pathways. Like most aGPCRs, mature ADGRG2 is a heterodimer resulting from cleavage at a highly preserved domain containing the GPCR proteolysis site (GPS) in an extracellular N-terminal fragment (NTF) non-covalently attached to a large C-terminal fragment (CTF) anchored in the cell membrane (Obermann et al. 2003). How these two sub-units cooperate under the action of endogenous agonists to mediate signals and do they have separate specific functions are questions that remain unanswered. However, it has been shown that the extracellular end of the CTF resulting from cleavage carries a *Stachel* sequence with agonistic properties (Demberg et al. 2015). In addition, recent experimental data obtained in in vitro and in vivo models show that via Gs and Gq protein-mediated signaling, ADGRG2, is capable of modulating c-AMP and PKC activity, respectively (Demberg et al. 2017; Balenga et al. 2016; Zhang et al. 2018).

### Causal mutations in CAVD, type, and epidemiology

#### *CFTR* mutations

Less than a year after the *CFTR* gene was identified (Riordan et al. 1989), Dumur et al. observed an abnormally high frequency of p.Phe508del mutation in a small series of infertile men with iCBAVD (Dumur et al. 1990). This discovery, which supported the hypothesis that iCBAVD might be a monosymptomatic form of cystic fibrosis, had a major medical consequence. Thereafter, all men with iCBAVD undergoing medically assisted reproduction technologies (ART) by surgical retrieval of sperm and intracytoplasmic sperm injection (ICSI) were to be considered to have a higher risk of having a child with cystic fibrosis (Anguiano et al. 1992). Subsequently, Chillon et al. confirmed that unlike CF patients who carry only CF-causing mutations responsible for a complete loss of CFTR chloride channel function, iCBAVD patients carry at least one *CFTR* copy with a so-called “mild” mutation, because it correlates with reduced or partial CFTR activity of 3–8% (Chillón et al. 1995). This situation is well illustrated by a variant of a polythymidine (Tn) polymorphism in intron 9 (NM_000493.3:c.1210-12T[5_9]), the so-called IVS8-5T allele (5T allele) whose frequency is four-to-five times higher in iCBAVD subjects (reviewed by De Sousa et al. 2018). This 5T allele has a deleterious effect on splicing which promotes the skipping of exon 10 leading to a significant reduction in normal *CFTR* mRNA (Chu et al. 1993). Up to one-third of iCBAVD subjects of European descent are compound heterozygotes carrying a CF-causing mutation, the most frequent being F508del, and the 5 T allele in *trans* (Chillón et al. 1995). However, since this genotype had been observed in fertile fathers who had a child with CF, Cuppens et al. showed that the penetrance of this 5 T allele with respect to the exon 10 skipping was mainly dependent on the size of a polyTG polymorphic sequence (NM_000493.3:c.1210-34TG[9_13]) upstream to the polyT sequence (Cuppens et al. 1998). Thus, while the polyvariant TG(11)5T (NM_000493.3:c.1210-34TG[11]T[5]) is found overwhelmingly in healthy subjects, it is the TG(12)5T combination that is most often found in iCBAVD subjects, while the much rarer TG(13)5T allele is always identified in iCBAVD subjects (Groman et al. 2004). Over the past 20 years, numerous studies have made it possible to characterize the *CFTR* mutation spectrum in CBAVD subjects by specifying their frequency according to ethnicity and geographical origins (reviewed by Yu et al. 2012). While the same types of severe mutations, including large *CFTR* rearrangements (Taulan et al. 2007), are found in both CF-CBAVD and iCBAVD subjects, the *CFTR* mutational spectrum in iCBAVD is radically different in that there are many non-CF-causing mutations, most of which can be associated with other CFTR-RD phenotypes such as pancreatopathies, disseminated bronchiectasis, and sinonasal disorders (Bombieri et al. 2011). These “mild” mutations mainly include intronic variants that affect splicing, the most frequent of which being the 5T allele, and numerous missense mutations that affect the functioning of the chloride channel, the most frequent in Caucasians being p.Arg117His (R117H) mutation (Casals et al. 2000; Claustres et al. 2000). Most of these non-CF-causing mutations are not detected by routine panels designed for the classic CF population, which mainly target the most frequent CF-causing mutations (numerous available commercial kits). That is why, for molecular diagnosis of CBAVDs and other CFTR-RDs, it is recommended to choose a *CFTR* test that includes the two main “mild” variants, R117H, and the 5T allele as a first-line test (see below the chapter “Implications for clinical practice …”). If this is inconclusive, a comprehensive characterization of *CFTR* should be performed, including at least sequencing of all exons and flanking intronic regions as well as a search for large rearrangements. Molecular diagnostic methods based on new-generation sequencing (NGS) are increasingly used for the detection not only of point mutations but also of large deletion or duplication. These new gene scanning methods which can be applied to a panel make it possible to avoid the laborious Sanger sequencing and semi-quantitative PCR techniques (MLPA, QMPSF, qPCR, etc.) carried out exon by exon.

The frequencies of *CFTR* mutations in CAVD patients differ from study to study, probably due to a recruitment bias, cohort size, and the heterogeneity of the genotyping methods, with many subjects having had a partial analysis of *CFTR*. However, it is clear that the frequency of some alleles is very different in Caucasian CAVD patients and those from non-Caucasian countries in which cystic fibrosis is much more infrequent. This is particularly the case with F508del mutation, which is exceptionally detected in Chinese iCBAVD patients, whereas up to one-third of the iCBAVDs patients in northern Europe are carriers. On the other hand, iCBAVD patients of Asian origin are more often carriers of the 5T allele than Caucasians (Table 1), while the frequency of this allele in the general population varies little around the world (5%). Overall, the meta-analysis of the data published by Yu et al (2012) indicates that approximately 80% of Caucasian iCBAVD patients are carriers of at least one mutation in *CFTR*. The most exhaustive study possible leaves 6% of the subjects without any detected mutation (Ratbi et al. 2007). Considering that some of these patients may be simple heterozygotes (3% in the Caucasian population) and others carry variants of unknown significance that are possibly neutral (not CF or CFTR-RD causing), it can be assumed that the *CFTR* would be implicated in 75–80% of the cases of iCBAVD. Therefore, for approximately a quarter of the iCBAVD patients, the responsibility of *CFTR* cannot be definitively proven, whereas for CF patients, the two mutated alleles can be characterized in 99% of the cases (Table 1). For CUAVDs, 30–50% of the subjects carry at least one *CFTR* mutation after comprehensive gene scanning, which means that more than half of the CUAVDs are not *CFTR-*related (Schlegel et al. 1996; Casals et al. 2000; Cai et al. 2019; Mieusset et al. 2020). The presence of a renal abnormality is very significantly more frequent in CAVD patients in whom only one or no *CFTR* abnormality has been detected (Augarten et al. 1994; Schwarzer & Schwarz 2012). Therefore, it can be assumed that the difference in the rate of non-detection of *CFTR* mutations between CBAVD (20%) and CUAVD (50%) is at least partially related to the difference in the frequency of unilateral renal agenesis observed in the two groups, 5% vs 25%, respectively (Weiske et al. 2000; McCallum et al. 2001; Kolettis and Sandlow 2002; Yang et al. 2015).

**Table 1:** Average frequencies of *CFTR* genotype classes according to CAVD forms with the most frequent alleles

	CF-CBAVD^a^	iCBAVD^b^	CUAVD^c^
CFTR genotypes			
[CF];[CF]	87%	0%	0%
[CF];[NCF]	11%	60%	15%
[NCF];[NCF]	< 1%	11%	5%
[CF or NCF];[O]	< 1%	16%	18%
[0];[0]	< 1%	13%	62%
More frequent [CF] allele			
Caucasian (North Europe)	[F508del] (70%)	[F508del] (28%)	[F508del] (15%) NA
Non-Caucasian (Chinese)	[G970D]^d^ (16%)	NA	NA
More frequent [NCF] allele			
Caucasian (North Europe)	NA	[5T] (20%) and [R117H] (5%)	[5 T] (30%)
Non-Caucasian (Chinese)	NA	[5T] (38%) and [Q1352H] (7%)	[5 T] (33%)^e^ and [Q1352H] (15%)

Percentages are calculated from compilations of data from large series (the main ones are referenced). These average frequencies are given as an indication to highlight differences related to ethnicity and CAVD forms

*[CF]* CF-causing allele, *[NCF]* non-CF-causing allele including mutations that result in a CFTR-RD and variations of unknown significance (VUS), *[0]* no pathogenic variants ([CF] or [NCF]) detected by full *CFTR* screening, *NA* non-applicable (various rare mutations)

^a^Claustres et al. (2017)

^b^Claustres et al. (2000)

^c^Cai et al. (2019); Mieusset et al. (2020)

^d^Tian et al. (2016)

^e^Yuan et al. (2019)

#### *ADGRG2* mutations

In 2016, after carefully selecting, from a large retrospective series of 379 iCBAVD men of European descent, a cohort of 26 individuals having neither *CFTR* mutation nor associated renal abnormality, Patat et al. identified three hemizygous truncating mutations in the X-linked *ADGRG2* gene (MIM#300572.0001_3) in four subjects (Patat et al. 2016). The establishment of the causal role of these mutations in the iCBAVD phenotype was based on a set of arguments: (i) male *ADGRG2* knockout (KO) mice develop OA without any other significant abnormality (Davies et al. 2004), (ii) histological examination of an epididymal biopsy of one of the four individuals showed a lack of expression of ADGRG2 in the epithelium of efferent ductules that were abnormally dilated, (iii) one of the truncated mutations was identified in two infertile individuals related by a maternal link (a nephew and a maternal uncle). Since then, three publications (Yang et al. 2017; Yuan et al. 2019; Khan et al. 2018) have reported the identification of five new rare variations of *ADGRG2* in six iCBAVD patients of Asian origin with no pathogenic *CFTR* mutation: two nonsense mutations classified as pathogenic, including one in two infertile brothers of Pakistani origin (Khan et al. 2018) and three missense mutations, including one affecting the GPS region which was classified as pathogenic (Yang et al. 2017). These six patients had no renal abnormalities. Recently, Pagin et al. has also reported six novel *ADGRG2* truncating mutations in a cohort of 53 French CAVD patients carrying 0 or only 1 *CFTR* defective allele. In this study, the authors failed to obtain convincing evidence to support the hypothesis of a digenic inheritance involving *ADGRG2* and *CFTR*. They concluded that inactivation of *ADGRG2* is responsible for approximatively 20% of CAVD not related to CFTR dysfunction. In addition, they found no case of solitary kidney among the 8 *ADGRG2* mutated patients of their cohort (Pagin et al. 2019). Interestingly, no *ADGRG2* or *CFTR* mutations were identified by Patat et al. in a cohort of 28 iCBAVD patients with URA (personal data).

#### Other mutations

To our knowledge, besides *CFTR* and *ADGRG2*, the only other mutations that have raised the question of a possible correlation with iCBAVDs are CNVs involving *PANK2* and *SLC9A3* genes. To date, these CNVs have only been described in iCBAVD patients from Taiwan. As in other Asian populations, CF and CFTR-RD are rarely observed in Taiwan and, apart from the IVS8-5T allele whose frequency is significantly increased in infertile Taiwanese iCBAVD men, very few pathogenic *CFTR* alleles have been characterized (Chiang et al. 2009). By investigating CNVs using array-CGH and quantitative real-time PCR in a small cohort of Taiwanese iCBAVD individuals, HS Chiang’s team identified in a single individual the homozygous loss of the pantothenate kinase 2 (*PANK*2) gene (Lee et al. 2009) and in 11 of 29 subjects, the loss of a copy of the solute carrier family 9 isoform 3 gene (*SLC9A3*) (Wu et al. 2018; reviewed by Chiang et al. 2019). *PANK2* was selected as a potential reproduction-related gene, because the KO mouse had azoospermia (Kuo et al. 2005). However, it was an NOA and this condition is not observed in affected humans with pantothenate kinase-associated neurodegeneration (MIM#234200). To date, no other cases of CBAVD related to a deletion of *PANK2* have been reported. Therefore, the correlation remains uncertain and the observation anecdotal. On the other hand, experimental data obtained by the same team on the involvement of *SLC9A3* in the CBAVD phenotype are substantial and more convincing. Indeed, these authors showed that the adult male mouse *SLC9A3-/-* develops obstructive azoospermia due to structural and functional abnormalities of the efferent ductules with long-term progressive atrophy of the vas deferens and seminal vesicles (Wu et al. 2019; Chiang et al. 2019). Remarkably, authors observed a drastic decrease in *CFTR* in the epididymis and vas deferens in these *SLC9A3*-KO mice suggesting interdependent roles of the two genes in iCBAVD determinism (Wang et al. 2017). However, despite these very enlightening observations on the role of *SLC9A3* in the physiology of the reproductive tract of male mice, the relationship between the loss of a copy of this gene and iCBAVD in humans is still poorly understood. Considering that the recessive mutations of *SLC9A3* cause a severe form of congenital diarrhea by sodium secretion (congenital secretory sodium diarrhea, MIM#616868) and that loss-of-function mutations, including a complete deletion of *SLC9A3*, have been shown to be transmitted by heterozygous fathers (Janecke et al. 2015), the CBAVD of Taiwanese patients cannot be explained by *SLC9A3* haploinsufficiency alone. Moreover, Wu et al. have reported in his study that among the 29 Taiwanese patients with iCBAVD, 6 (20.7%) were found homozygous or compound heterozygous for the *CFTR* TG(12)5T or TG(13)5T alleles, a genotypic status that could be sufficient on its own to cause iCBAVD. Of these six individuals, two had only one copy of *SLC9A3*. From this same cohort, 12 other iCBAVD patients were heterozygous for the TG(12)5T or TG(13)5T allele, half of whom also had a deletion of *SLC9A3* (Wu et al. 2019). The possibility of digenism involving *CFTR* variations such as the 5T allele is still highly speculative. It should be noted that none of these 29 Taiwanese CBAVD patients had a unilateral renal absence. 

## Physiopathology: atresia or agenesis?

The pathophysiological processes that lead to CAVD are still widely misunderstood. However, all clinical and fetal observations, as well as clinical–genetic correlations and experimental data from KO animal models accumulated over the last three decades, have led to the emergence of two major pathophysiological concepts. The first postulates that it is the loss of homeostasis (water–ions–pH) of the intraluminal fluid due to the dysfunction of the seminal duct epithelium that is the cause of a progressive involution of the VDs, a concept modeled on CF. The second concept postulates that it is an early organogenesis disorder that leads to an absence of VD development, an hypothesis which is based on the fact that a significant proportion of CAVDs are accompanied by the unilateral absence of a kidney (URA). These two concepts could apply to two distinct populations of CAVD men: one that combines a high frequency of mutations in *CFTR* or *ADGRG2* and a low frequency of URA association, and one that combines the same criteria but with respectively inverted frequencies. Data from well-phenotyped CAVD cohorts with sufficient size indicates that these two populations do exist: URA is rarely observed in men with CF (CF-CAVD), while, on the other hand, *CFTR* or *ADGRG2* mutations are rarely identified in iCAVD men with URA (Patat et al. 2016; Pagin et al. 2019).

In CF-CAVD subjects, the hypothesis of VD regression due to obstruction by intraluminal mucus accumulation was initially suggested by simple analogy with lesions observed in other exocrine systems, particularly the pancreas (Di Sant'Agnese 1968). It was then taken up again after the secretory function of CFTR was understood. The absence of expression or a significant reduction in activity of the CFTR protein which affects the transepithelial exchanges of ions and water might be responsible for an increase in the viscosity of the fluid from the epididymis and vas deferens until the lumen is obliterated, which could induce degenerative lesions of the ducts (Patrizio and Zielenski 1996). This hypothesis of progressive obstruction was supported by several elements: (i) the expression of *CFTR* along the male excretory tractus, (ii) the high secretory activity, and (iii) the slow flow rate in a very coiled epididymis lumen. In addition, several observations of CF male fetuses at a gestational age of 12–22 weeks who had a morphologically normal tract showed that CF-CAVD does not result from agenesis but from a pathophysiological process that probably begins after the first trimester of gestation and continues after birth (Gaillard et al. 1997; Marcorelles et al. 2012). Degenerative lesions that gradually lead to VD atresia and then to their disappearance might occur mainly in prepubescent children, as shown by the first autopsy reports of CF children (Oppenheimer and Esterly 1969; Valman and France 1969). However, this first hypothesis implied that duct atrophy would be a direct result of lumen obstruction, which, so far, has never been proven either in fetuses or experimentally (Gaillard et al. 1997; Plyler et al. 2019). This pathogenesis remains speculative and is still based on an extrapolation of the pathophysiology of the CF pancreas. However, this analogy is not very relevant because of the difference in the composition of the secretions of the two tracts. While exocrine pancreatic secretion is mainly composed of proteolytic enzymes that are potentially harmful to epithelial integrity, seminal duct secretion is of a fundamentally different nature. Several recent studies based on animal CF models (*CFTR−/−*) have invalidated the hypothesis of a process initiated by mucus accumulation. Therefore, neither in CF pigs (Pierucci-Alves et al. 2011) nor in CF rats (Plyler et al. 2019) were traces of PAS-positive mucosal plugs or signs of obstruction in the normally developing genital tract observed. On the other hand, in the recent study by Plyler et al., *CFTR−/−* neonatal rats showed significant structural alterations in some portions of the vas deferens and epididymal duct, including underdevelopment of the periductal smooth muscle and disorganization of the adjacent epithelium. These lesions progress rapidly after birth and, within a few days, lead to the underdevelopment of the epididymis and collapsed VDs which eventually disappear or become atretic in mature CFTR−/− rats (Plyler et al. 2019).

**Fig. 5:**
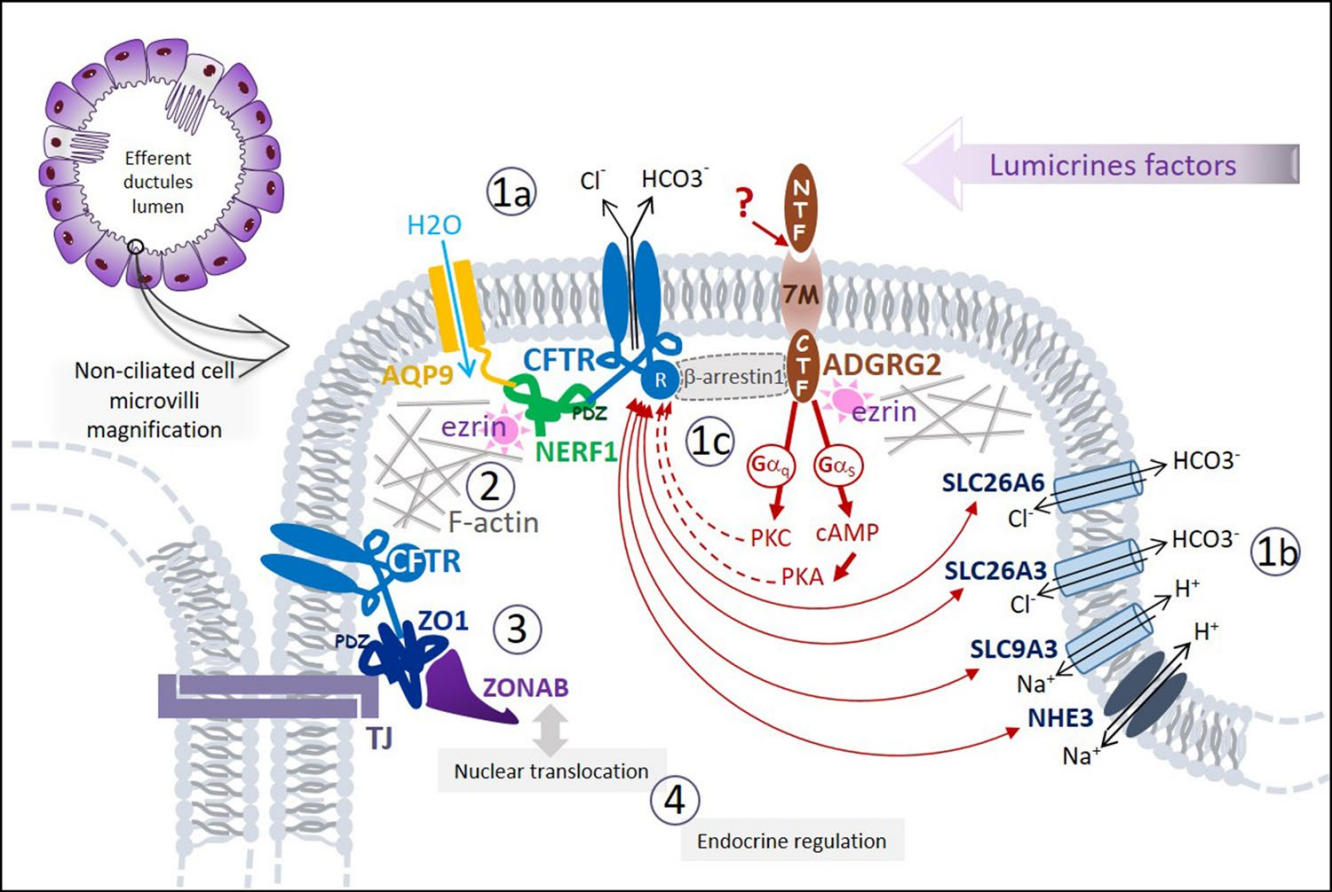
Representation of an enlarged non-ciliated cell microvilli with a schematic overview summarizing different molecular models* of four pathophysiological processes induced by deleterious mutations of *CFTR*, *ADGRG2*, or *SLC9A3*: impairment of the intraluminal fluid water–ion–pH homeostasis (1) by at least one of the following three mechanisms, (i) deregulation of the water channel c-AMP dependent aquaporin-9, AQP9 (1a); (ii) deregulation of the apical electrolytes transport and the intraluminal pH by impairment of the complex interaction networks linking the co-localized Cl^−^ channel CFTR, Cl^−^/HCO3^−^ exchangers (SLC26A3/A6) and Na^+^/H^+^ exchangers (NHE3, SLC9A3) (1b). (iii) deregulation of the luminal fluid reabsorption by ADGRG2-activated Gq protein deficiency (1c); deregulation of CFTR and/or ADGRG2 intracellular trafficking (2); impairment of the epithelial integrity and tubulogenesis by tight-junctions’ (TJs) disorganization (3); impairment of the epithelial cell proliferation/differentiation regulation (4). *Almost of these models has been constructed from experimental data of in vitro or in vivo studies with knockout animals (mouse or less often rat)

While in the adult male tract, the level of CFTR expression is highest in the epithelium of the epididymis head where 90% of fluid reabsorption occurs, in male fetuses, CFTR expression, detected as early as in 20 weeks of gestation, is homogeneous throughout the epididymis and vas deferens, suggesting that CFTR has different functions during development (Marcorelles et al. 2012). The spatial and temporal regulation of CFTR expression during development might depend on lumicrine factors and androgens (Breton et al. 2016). Recent findings have shown that *CFTR* is involved in regulating the proliferation and differentiation of male tract epithelial cells. The first evidence concerns the relationship with Wnt/β-catenin signaling pathway which is involved in WD differentiation (Carroll et al. 2005). RNAseq and immunohistochemical analysis of male reproductive cord of newborn CFTR−^/^− rats showed that the expression of Wnt9b in the epididymis was strongly diminished or even undetectable compared to control neonates (Plyler et al. 2019). Another evidence concerns the regulation of the tight junction (TJ) complexes that are essential for the integrity of epithelial cells and the formation of tubular structures. Ruan et al. have shown that CFTR is able to interact in vitro via its PDZ-binding domain with the ZO1/ZONAB complex of TJ of the epididymal epithelium (Ruan et al. 2014). In the Cftr−/− mouse, the decrease in *CFTR* expression in the epididymis results in a reduction in ZO1 expression and ZONAB translocation in the nucleus that induces a decrease in differentiation and an increase in epithelial proliferation (Fig. 5). Other studies have supported the hypothesis that VD degeneration is largely due to a morphogenetic and differentiation disorder of male tract epithelial cells. Thus, SLC9A3^−/−^ mice exhibit a cytoarchitectural disorganization of the epididymal duct and vas deferens that gradually worsened while the expression of CFTR is drastically reduced (Wang et al. 2017). These authors also showed that SLC9A3 and CFTR are co-located in the non-ciliated cells of mouse epididymis and that their expression is interdependent. However, how these two proteins interact remains unexplained. A recent functional study showed that SLC26A3 another anion exchanger expressed in the genital tract is able to activate CFTR by attaching itself via its STAT domain to the R domain of CFTR (Fig. 5). This activation is suppressed by a missense mutation in the STAT domain, the frequency of which is significantly increased in infertile men of Finnish origin (Wedenoja et al. 2017). In addition, in male SLC26A3^−/−^ mice, fertility is reduced mainly due to severe disorganization of epididymal cytoarchitecture (El Khouri et al. 2018).

Since the loss-of-function mutations of *ADGRG2* are responsible for an X-linked iCBAVD phenotypically similar to a CF-CBAVD, it has been assumed that CFTR and ADGRG2 may be involved in a common pathophysiological mechanism (Patat et al. 2016). Kirchoff et al. showed that there is a high expression of ADGRG2 in the microvilli of non-ciliated cells of the efferent ductules where it co-locates with the F-actin-ezrin scaffold (Kirchhoff et al. 2008). CFTR activity at the apical membrane also depends on interactions with the actin cytoskeleton mediated by the scaffolding protein NHERF1 and the actin-binding protein ezrin (Sun et al. 2000; Edelman 2014). Thus, CFTR C-terminus is able to bind the NHERF1 PDF domain that promotes formation of a CFTR–NERF1–ezrin–actin complex (Seidler et al. 2009; Pietrement et al. 2008) (Fig. 5). Recently, it has been shown that in the epithelium of the efferent ductules of male mice, CFTR forms an apical complex with ADGRG2 and β-arrestin-1 (Zhang et al. 2018). These experimental data suggest that ADGRG2, whose endogenous ligand is unknown, activates CFTR and regulates the fluid reabsorption through Gs-cAMP-PKA and Gq-PKC signaling and an arrestin-1-mediated ADGRG2/CFTR coupling (Fig. 5). Thus, the absence of ADGRG2 would prevent the formation of this regulatory complex and might cause the dilation of the efferent ductules by the accumulation of fluid.

In summary, CFTR interacts via PDZ-scaffolding proteins with many actors involved in regulation of the water channel (c-AMP-dependent aquaporin-9), ionic transfers (SLC26A3, SLC26A6 and SCL9A3), cytoskeletal organization, (ezrin) or signaling (ADGRG2, Wnt9b, ZONAB). All these partners work in synergy thanks to interactions with PDZ protein networks and with the prominent CFTR regulator (R domain) (Kujala et al. 2007) (Fig. 5). The formation at the apical membrane of these stable multiprotein complexes is crucial for the morphogenesis and maintenance of the epithelial integrity of male excretory pathways during fetal and/or postnatal life. This could explain why the absence or dysfunction of one of these molecules can ultimately lead to a degeneration of the vas deferens, via various pathophysiological mechanisms, as summarized in Fig. 5. Moreover, endocrine (androgens and estrogens) and paracrine (lumicrine) factors are involved in these different pathophysiological process induced by CFTR, ADGRG2, or SLC9A3 dysfunction contributing in particular to the timing of the progressive deterioration of the reproductive tractus (Plyler et al. 2019; Breton et al. 2016).

Unlike CFTR-related CAVDs, for which numerous studies on *CFTR* functions have been conducted, the pathogenesis of CAVDs without *CFTR* or *ADGRG2* mutations has hardly or never been explored. The frequent association of these unmutated CAVDs with renal abnormalities, particularly URA, has been reported in numerous studies suggesting an etiopathogenesis that is different from that of CF-CBAVDs. This situation is particularly striking for CUAVDs where the ipsilateral absence of a kidney is found in at least a quarter of the reported cases (Donohue and Fauver 1989; Schlegel et al. 1996). In both sexes, URA is frequently accompanied by genital abnormalities and is presumed to result from an embryonic development disorder occurring in approximately the 5th week of gestation (Shapiro et al. 2003). The embryonic anomaly may involve not only the ureteric bud but also the other derivatives of Wolff's canal including the seminal vesicles, vas deferens, and epididymis. Apart from a few multi-organ syndromes with which URA can be associated, no genetic basis is known to date and isolated familial forms of URA have rarely been reported (Woolf and Hillman 2007). The determinism of URA-CAVDs is probably complex depending on genetic, epigenetic, and environmental factors. It is perplexing that URA-CAVD is more rarely reported in studies of non-European populations that are actually well documented (Yang et al. 2015; Radpour et al. 2008; Sharma et al. 2014; Wu et al. 2004; Yuan et al. 2019). New studies using integrative multi-omics approaches will probably make it possible to specify the share of determinism linked to genetic mutations and to identify possible epigenetic causes of URA-CAVDs in distinct ethnic populations of origin.

## Implications for clinical practice and genetic counseling

**Fig. 6:**
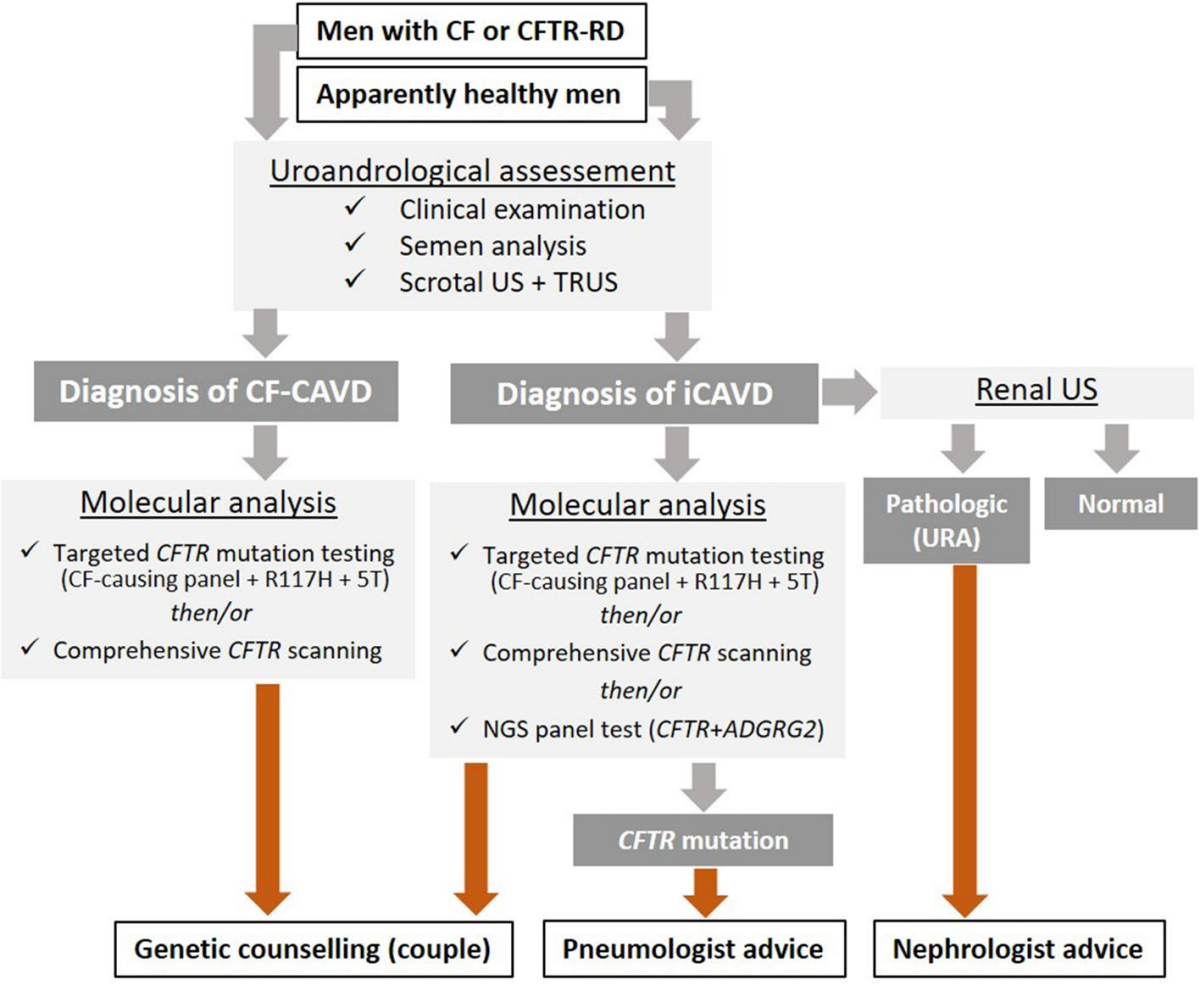
Flowchart for managing men referred to the andrologist for suspected CAVD. Uroandrologic assessment should include clinical examination (anamnesis and scrotal palpation), semen analysis (sperm count, pH, volume, and biochemical markers), and a scrotal ultrasonography (US) plus a transrectal ultrasonography (TRUS). This assessment is mandatory to confirm and characterize any suspicious CAVD (Mieusset et al. 2020). *URA* unilateral renal absence

**Fig. 7:**
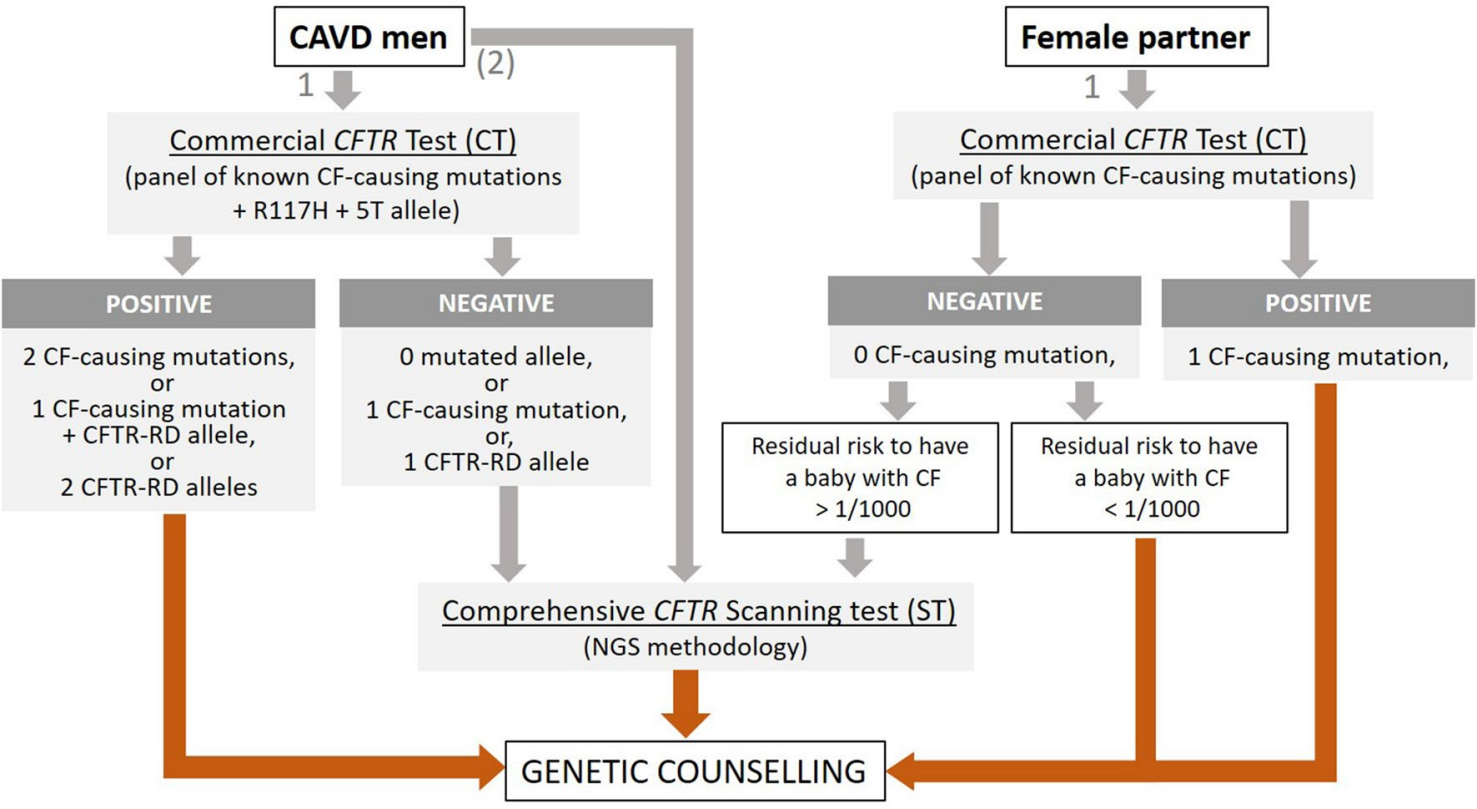
Flowchart of the *CFTR* testing process for genetic counseling purposes in couples where the male partner has a CAVD. 1: first choice strategy, that consists in carrying out a *CFTR* targeted CF-causing mutation test (commercial kit) in the two partners. Because for men with isolated CAVDs, particularly CUAVDs, the commercial *CFTR* test is often negative, it may be appropriate, as an alternative strategy (2), to directly apply a *CFTR* scanning method

CAVD is a relatively rare condition, but its diagnosis has beyond the problem of infertility, important other medical implications for the patient because of its frequent association with renal anomalies and *CFTR* mutations. These two parameters should not be omitted from the diagnostic process once a CAVD has been correctly characterized, because they have important medical consequences for the patient and for his family. A flowchart for the medical management of the following two categories of subjects suspected of having CAVD is described in Fig. 6. On one hand, young adults with medical follow-up for CF or CFTR-RD are informed of their high probability of sterility, and so they are referred to the andrologist to confirm the CBAVD and to benefit from assisted reproduction technologies (ART) using surgical sperm retrieval and in vitro fertilization techniques. On the other hand, an isolated CAVD (iCAVD) can be discovered by chance or because of infertility in an “a priori” healthy man. A significant proportion of these individuals carry pathogenic *CFTR* mutations and some of them may express mild cystic fibrosis symptoms such as recurrent respiratory tract infections with the risk in the future of developing serious complications (disseminated bronchiectasis, chronic pancreatitis, diabetes, etc). Thus, evaluation of these men with iCAVD should include careful clinical examination and *CFTR* analysis. The most suspicious cases, especially those carrying a CF-causing mutation, should be referred to a pneumologist of a CF care center for specialized management and follow-up (Fig. 6). However, the most common comorbidity of CAVD is probably the association with the other defects of the urogenital system, notably the solitary kidney (URA) which was found in about one-third of the CUAVD cases (Akinsal et al. 2018; Mieusset et al. 2020). Thus, an abdominal ultrasound for renal imaging is strongly recommended for all men with iCAVD (Fig. 6). If a solitary kidney is detected, patients should be counseled about the increased risk of renal anomalies in their offspring (Kolettis and Sandlow 2002; McCallum et al. 2001). Last but not least, genetic counseling should be given to all men with CAVD who wish to have a child with their own gametes (Figs. 6, 7).

The medical management of a man with known or suspected CAVD must include *CFTR* analysis, given the high frequency of mutations in this gene associated with this condition. In fact, *CFTR* genotyping has a dual importance for these men: (i) it clarifies the clinical diagnosis on a molecular basis and sheds light on the pathophysiological mechanism; (ii) it refines the risk, a priori increased for these men, of carrying a CF-causing mutation, in other words, the risk for himself and for their relatives to develop a CFTR-RD and/or to transmit CF to the offspring. This risk is higher when the CAVD is bilateral and without association with URA (2/3 vs 1/30, NT in Table 2). *CFTR* genotyping in men with CAVD should include, at a minimum, targeted research for the most common CF-causing mutations as well as R117H and the 5T allele (Fig. 6). This targeted and inexpensive strategy, easily accessible thanks to the use of available commercial kits, is convenient as a first approach, because an unambiguous genotypic diagnosis is often obtained, especially in the case of iCBAVD and when the subject is of European descent (recurrent genotypes including a CF allele such as F508del associated with a CFTR-RD allele such as R117H or allele 5T). However, due to the high number of rare CFTR-RD alleles and non-Caucasian ethnicities, comprehensive scanning of *CFTR* for rare-point mutations and large rearrangements may be necessary for all cases of CAVD with no or a single mutation identified by the targeted test. A recent methodological evolution which tends to be generalized is not to use in the first approach *CFTR* mutation-targeted tests but to directly offer a comprehensive scanning by NGS methodology of a panel of genes including *CFTR*, *ADGRG2* and other genes linked to male infertility. We consider that if this strategy can be beneficial for exploring symptomatic subjects such as men with a confirmed CAVD (Fig. 6), on the other hand, it is not appropriate and is potentially harmful in the context of genetic counseling.

**Table 2:**
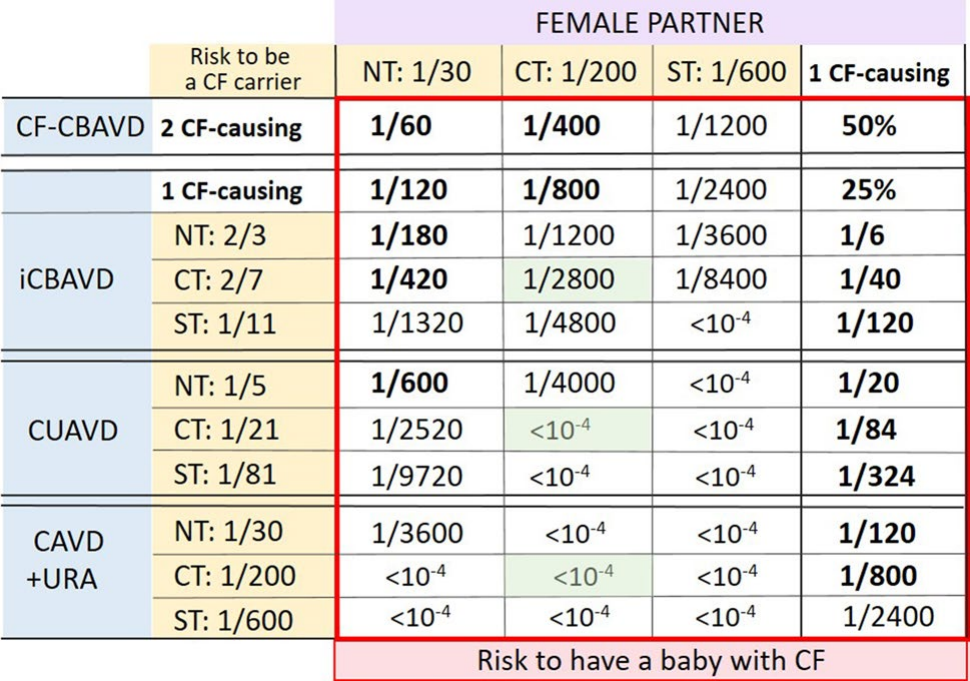
Selected examples of a priori risks (NT) and Bayesian probabilities after *CFTR* testing (CT and ST) to be a CF carrier according to the type of CAVD, with resulting calculations of the risk for the couple in having a CF child

It is emphasized that the frequency of CF-causing mutations, and therefore, the sensitivity of screening tests strongly depends on the ethnicity and geographical origins of individuals. The table values are given as examples for individuals of European descent who have an a priori risk (before any testing, NT) of 1/30 (Farrell 2008) and for whom the average sensitivity of the *CFTR* mutation screening tests is 85% (routine mutation panel test: CT) or 95% (comprehensive scanning test: ST)

NT: no *CFTR* test means a priori risk; CT: commercial *CFTR* kit for the search of the most common CF-causing mutations (usually 50 mutations) giving a 85% sensibility (for Caucasian populations); the indicated value is the Bayesian residual risk to be a CF carrier if the test is negative; ST: comprehensive scanning *CFTR* test (whole-exon/flanking sequencing) giving at least 95% sensibility; the indicated value is the Bayesian residual risk to be a CF carrier if the test is negative.

All probability values about the risk to be a CF carrier are indicated in the table on a yellow background. All probability values about the risk for the couple in having a baby with CF are indicated in the red square. Values represented in bold in the red square mean a risk higher than 1/1000

*CF-CBAVD* CF patient with bilateral CAVD, *iCBAVD* patient with isolated bilateral CAVD, *CUAVD* patient with unilateral CAVD, *CAVD + URA* patient with CAVD and unilateral renal absence. The CF-causing mutation frequencies used for probability calculations are those indicated in Table 1.

Appropriate genetic counseling is strongly recommended for any man with a CAVD who plans to have a child with his own gametes (natural fertilization or via ART). Since the primary objective of this genetic counseling is to assess the risk for the couple to have a CF child, ideally, it should be provided to both partners prior to conception. In practice, the most efficient way is to offer to the couple a genetic testing for CF-causing mutations (for the flowchart, see Fig. 7). Commercially available *CFTR* kits which target the most common CF-causing mutations (CT, in Table 2) are routinely used providing quick and limited-cost result, but limitations of these tests in terms of sensitivity must be explained, so that the couple understands the notion of residual risk. If the test is negative for both partners, the risk of the couple having a CF child is greatly reduced (< 1/1000, green square in Table 2). If the test is positive for one of the two partners, based on Bayesian probabilities, the residual risk may be high enough (> 1/1000, bold values in Table 2) to justify, in a second step, a complementary *CFTR* analysis (scanning test with whole exons/flanking sequencing, ST in Table 2) for the one whose test is negative. This second test is strongly recommended if the female partner with a negative result for the most frequent CF-causing mutation is not of north European descent (low sensitivity of the commercial kit) or if her partner with CAVD has CF (2 CF-causing mutations). However, it should be emphasized that the drawback of this comprehensive scanning method is the risk of identification of non-CF-causing mutation and *CFTR* variations of unknown significance making genetic counseling very challenging. If the first test is positive for both partners, the risk of the couple having a child with cystic fibrosis is either 50% (partner with cystic fibrosis) or 25% (Table 2). In this situation, information on the different reproductive options (prenatal diagnosis, pre-implantation diagnosis, etc.) must be provided to the couple to avoid the birth of an affected child. If the CAVD man has no mutation after comprehensive *CFTR* testing and normal renal imaging, an *ADGRG2* analysis should be suggested, especially if there is a family history of male infertility.

## Conclusion

CFTR-related CBAVD are the most well-known forms of CAVD because of their involvement in male infertility and their association with CF-causing mutations. That is why, they were the most studied. Thus, numerous studies carried out over the past 3 decades has led to an exhaustive characterization of the CAVD-causing *CFTR* variations and the highlighting of the diversity of their spectrum according to geographical and ethnic origins. At the same time, consistent experimental data obtained from relevant animal models such as the rat or the pig have shown that the CBAVD induced by CFTR dysfunction results more likely from a gradual involution initiated at the beginning of the third trimester of pregnancy and continuing after birth, rather than a simple developmental defect due to an obstruction by a viscous fluid, as originally believed. By studying these animal models, authors have begun in recent years to describe the complex CFTR-dependent processes which are crucial for the maintenance of fluid homeostasis and the integrity of the epithelium of the male seminal tract. First findings have led to the emergence of several pathophysiological hypotheses that are still not validated in human. Other genes contribute to these processes such as the *ADGRG2* whose mutations cause a minority of CBAVD not linked to *CFTR* variations. The discovery of these genes and the study of their role in the maintenance of the male seminal tract are essential to understand the pathophysiological mechanisms that lead to the atresia of the vas deferens. However, while the genetic heterogeneity of CAVD has recently been confirmed, it probably does not explain all the phenotypic diversity. Thus, the hypothesis that the frequent association of CUAVD and solitary kidney is not solely caused by genetic variations has not so far been adequately explored. What role might play endocrine, epigenetic, or environmental factors in the determinism of these forms of CAVDs, that is an issue that poses a significant challenge for future research on this topic.

“The second lesson to be learnt is that sterility is occasionally the result of some pathological condition of the vas the nature of which is still obscure. It is to be hoped that further research will throw light on this interesting problem.” John Hunter in 1775.
